# Intercostal Cryoanalgesia in Pediatric Nuss Repair for Pectus Excavatum: A Three-Case Series From Ecuador

**DOI:** 10.7759/cureus.96105

**Published:** 2025-11-04

**Authors:** Juan Esteban Cruz Álvarez, Jose Antonio Daza Merizalde, Michele José María Ugazzi Betancourt, Giancarlo Daniel Sánchez Salazar

**Affiliations:** 1 Pediatric Surgery, Universidad San Francisco de Quito, Quito, ECU; 2 Pediatric Surgery, Hospital Pediátrico Baca Ortíz, Quito, ECU

**Keywords:** intercostal cryoanalgesia, low-resource settings, nuss procedure, pectus excavatum, pediatric thoracic surgery, postoperative pain management

## Abstract

Pectus excavatum is the most common congenital anterior chest wall deformity, and although the Nuss procedure is the standard of care, postoperative pain remains a clinical challenge. Here, we report a three-case series from Quito, Ecuador, evaluating ultrasound-guided intercostal cryoanalgesia integrated with pediatric Nuss repair. Three adolescent males (14-15 years) with moderate-to-severe deformity (mean Haller index: 3.51) underwent two-bar Nuss repair with lateral stabilizers. Bilateral intercostal cryoanalgesia (T3-T8; two minutes per nerve; CO₂ cryoprobe) was performed at three timings: intraoperative (day zero), immediate preoperative (-24 hours), and delayed preoperative (-90 days). A standardized multimodal protocol included bilateral pectoralis nerve II blocks, a type of ultrasound-guided regional anesthetic technique used to manage chest wall pain, plus a dexmedetomidine infusion and scheduled non-opioid analgesics; tramadol was reserved for rescue via the intravenous route. There were no intraoperative complications or intensive care admissions. Pain decreased rapidly, as measured by the Visual Analog Scale, with day one scores of 2-3 and day three scores of 1-2; opioid rescue was minimal (0-1 dose within 24-48 hours), and the length of stay was short (3-4 days). No neurologic deficits or wound complications were observed. These observations support the feasibility and safety of intercostal cryoanalgesia in a middle-income setting and suggest that immediate preoperative application (-24 hours) may provide earlier analgesic onset than intraoperative timing, warranting larger comparative evaluations.

## Introduction

Pectus excavatum is the most prevalent anterior chest wall deformity in children and adolescents [1]. The minimally invasive Nuss procedure, a technique involving the sub-sternal placement of a concave steel bar to correct chest depression, has become the standard surgical approach [2,3]. A case series on thoracoscopic cryoanalgesia has been reported from Latin America [4]. Nevertheless, substantial postoperative pain may delay ambulation and prolong the length of stay [5].

Intercostal cryoanalgesia, a regional anesthesia technique that induces reversible axonal injury while preserving the epineurium, has been shown to be associated with reduced opioid requirements and shorter hospitalization in contemporary cohorts and a randomized trial [6-9]. Despite growing adoption in high-resource centers, experience from low- and middle-income countries (LMICs) remains limited owing to equipment availability, training, and cost barriers [10].​​​​​​​

Given that pain management is a major barrier to early recovery [5] and that over 90% of patients with moderate-to-severe deformities undergo surgery primarily for cosmetic or psychosocial reasons [1], optimizing perioperative pain control is crucial in adolescent populations with pectus excavatum.

## Case presentation

We present three individual cases of otherwise healthy adolescent males (ages 14-15 years) who underwent minimally invasive Nuss repair for moderate-to-severe pectus excavatum in Quito, Ecuador. Indications were primarily cosmetic; no clinically significant cardiopulmonary impairment was observed on preoperative assessment (spirometry and echocardiography were normal in all patients). Medical histories were unremarkable, with no chronic medications or allergies. All procedures were elective, with informed consent obtained from patients’ legal guardians.

Pectus excavatum was symmetric in all three patients. Associated symptoms included occasional chest pain and exertional fatigue, but no severe dyspnea or exercise intolerance was documented. The Haller index, a radiologic severity measure calculated as the transverse diameter of the chest divided by the anteroposterior distance, ranged from 2.5 to 4.4, consistent with surgical indication in all cases.

Each patient received bilateral ultrasound-guided intercostal cryoanalgesia targeting T3-T8 intercostal nerves with a CO₂ cryoprobe at -60°C to -80°C for two minutes per level. The timing of cryoanalgesia differed: intraoperative (day zero), immediate preoperative (-24 hours), and delayed preoperative (-90 days). Standardized surgical technique involved the placement of two retrosternal bars and lateral stabilizers. All patients followed the same multimodal analgesia protocol: bilateral pectoralis nerve (PECS) II blocks (bupivacaine 0.25%), intravenous (IV) dexmedetomidine infusion, and scheduled non-opioid analgesics. Tramadol IV was reserved for rescue if the Visual Analog Scale (VAS) score was >3/10.

Case 1: Intraoperative cryoanalgesia (day zero)

A 15-year-old male with a Haller index of 3.63 underwent Nuss repair with bars at the fourth and sixth intercostal spaces. Intraoperative cryoanalgesia was performed bilaterally at T3-T7. Anesthesia included lidocaine 60 mg IV, rocuronium 30 mg, and dexamethasone 8 mg. Additional medications included paracetamol 1 g, ketorolac 30 mg, and ondansetron 9 mg. Pain scores on VAS were 9 (hour six), 3 (day one), and 1 (day three). One dose of tramadol (100 mg IV) was required. The patient ambulated within 24 hours and was discharged on day three. No complications were observed. Follow-up at three months confirmed complete pain resolution and normal daily activities.

Case 2: Preoperative cryoanalgesia (-24 hours)

A 15-year-old male with a Haller index of 4.40 underwent repair with bars at the fourth and fifth intercostal spaces. Cryoanalgesia was performed 24 hours before surgery at T3-T8 bilaterally. Induction included lidocaine 60 mg IV, rocuronium 40-45 mg, dexamethasone 8 mg, and midazolam 1 mg. Additional medications included paracetamol 1 g, ibuprofen 600 mg, morphine 7 mg, and a second dose of dexamethasone 8 mg. VAS scores were 9 (hour six), 2 (day one), and 1 (day three). No opioid rescue was needed. Independent ambulation occurred within 48 hours, and discharge was on day three. At the three-month follow-up, no residual symptoms were reported.

Case 3: Preoperative cryoanalgesia (-90 days)

A 14-year-old male with a Haller index of 2.50 received cryoanalgesia 90 days before repair, at T3-T8 bilaterally. Bars were placed at the fifth and seventh intercostal spaces. Anesthesia included midazolam 1-3 mg, fentanyl 50-100 μg, lidocaine 60 mg IV, propofol 80 mg, and rocuronium 45 mg. Adjuncts included dexamethasone 8 mg, tranexamic acid 500 mg, magnesium sulfate 1.2 g, morphine 7 mg, paracetamol 750 mg, metamizole 1 g, and ketorolac 40 mg. VAS scores were 9 (hour six), 2 (day one), and 2 (day three). One dose of tramadol (100 mg IV) was required. The patient was discharged on day four without complications. At follow-up, the patient reported adequate pain control and had resumed full physical activity.

**Figure 1:**
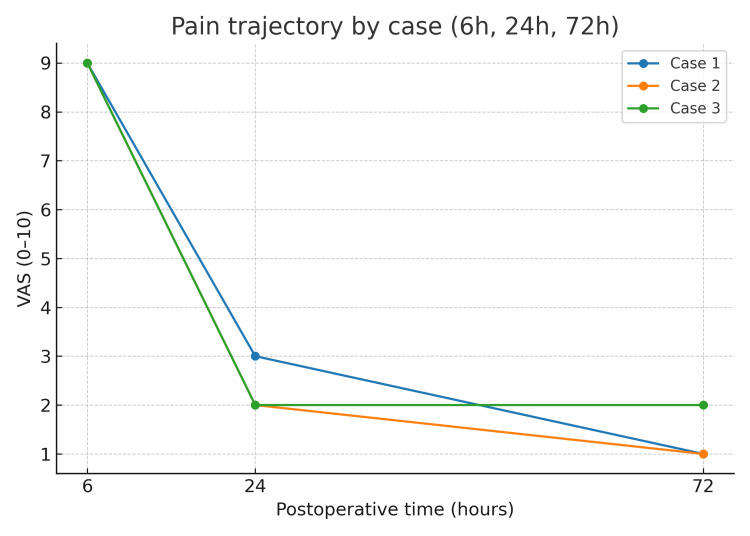
Pain trajectory (VAS 0–10) at 6, 24, and 72 hours after Nuss repair with intercostal cryoanalgesia. Lines represent individual cases according to cryoanalgesia timing: Case 1—intraoperative (day zero); Case 2—preoperative (-24 hours); Case 3—preoperative (-90 days). Pain was measured using the VAS (0-10). Error bars are omitted because only single observations were available per time point. VAS = Visual Analog Scale

Pain scores across the first 72 hours are shown in Figure 1, with a rapid decline from a VAS score of 9 at 6 hours to a VAS score of 1-2 by postoperative day three in all cases. A detailed comparison of demographics, cryoanalgesia timing, opioid rescue, and clinical outcomes is presented in Table 1.

**Table 1:** Demographics, cryoanalgesia timing, and immediate postoperative outcomes in three pediatric patients undergoing Nuss repair. This table summarizes clinical demographics, Haller index, surgical technique, timing of intercostal cryoanalgesia, length of stay, opioid rescue requirements, and postoperative pain scores (VAS) in the first three pediatric cases treated in Ecuador. ICS = intercostal space; LOS = length of stay; VAS = Visual Analog Scale (0–10)

Case (sex, age)	Haller index	Bars (ICS levels)	Cryo timing (levels)	LOS (days)	Opioid rescue (24–48 hours)	VAS score day 1/day 3
Male, 15	3.63	2 (4th and 6th)	Intraoperative; T3–T7 bilateral	3	1 dose	9/1
Male, 15	4.40	2 (4th and 5th)	Preoperative -24 hours; T3–T8 bilateral	3	0 doses	9/1
Male, 14	2.50	2 (5th and 7th)	Preoperative -90 days; T3–T8 bilateral	4	1 dose	9/2

The ultrasound-guided cryoanalgesia protocol used in all patients, including positioning, marking, puncture technique, and real-time ice-ball formation, is demonstrated in Video 1.​​​​​​​

**Video 1:** Ultrasound-guided intercostal cryoanalgesia during pediatric Nuss repair.

No adverse neurologic effects such as paresthesia, numbness, or sensory loss were reported in any of the three patients during hospitalization or at follow-up. This supports the known safety profile of cryoanalgesia in preserving intercostal nerve function by avoiding irreversible axonal injury.

## Discussion

This report demonstrates that intercostal cryoanalgesia can be implemented safely and effectively in an LMIC as part of multimodal analgesia for pediatric Nuss repair, yielding rapid pain reduction, minimal opioid exposure, early ambulation, and short hospitalization. In our three cases, pain decreased from a VAS score of 9 at six hours to a VAS score of 1-2 by day three, with only 0-1 dose of opioid rescue and a hospital stay of 3-4 days. Our findings are consistent with a randomized clinical trial showing reduced opioid use and shorter length of stay with intraoperative cryoablation versus conventional strategies, and with observational series reporting similar benefits [6,9].

Compared to conventional analgesia strategies such as epidural catheters or IV opioids, intercostal cryoanalgesia has demonstrated lower opioid requirements, reduced ICU admission rates, and improved ambulation in the early postoperative phase [4,6,7]. In our setting, no adverse sensory symptoms (numbness, dysesthesias, or neuropathic pain) were observed during follow-up. This absence of neurologic events aligns with the expected safety profile and is clinically relevant for assessing nerve-related risks.​​​​​​​

Regarding timing, randomized and comparative cohorts consistently demonstrate reduced length of stay and opioid use with intercostal cryoanalgesia overall [6,9]. The physiologic rationale for a -24-hour application is that the sensory block typically matures over ~24-36 hours [11], so performing cryo the day before surgery aligns peak blockade with the early postoperative pain window and may outperform same‑day cryo in the first 24-48 hours. Adoption is not universal because of practical trade‑offs: additional operating room time to treat multiple intercostal levels (≈25-30 minutes in pediatric series [11]) and, in the randomized controlled trial by Graves et al., +46.5 minutes vs. thoracic epidural [7]); equipment and disposable probe costs (a multicenter analysis reported higher total cost versus intravenous patient-controlled analgesia, US dollar (USD) $11,145 vs. $8,975, driven largely by operating room supplies [10]), although other studies have found lower overall resource utilization with cryo [8]; and transient chest wall hypoesthesia, which typically resolves by 6-12 months, with persistent numbness or neuropathic pain being uncommon [8,12]. Early prospective data also suggest that one-minute freezing per level is non-inferior to two minutes, which could mitigate time and cost [13]. No cryo-related adverse events occurred, aligning with published safety profiles.​​​​​​​

In LMICs, additional barriers include limited access to cryoprobe equipment, lack of trained personnel, and unfamiliarity with intraoperative cryotechnology. Despite these limitations, the successful implementation in our center illustrates the feasibility. A prior Latin American report [4] also described thoracoscopic cryoanalgesia as a novel adjunct, though without ultrasound guidance or preoperative timing. This comparison highlights our approach as a complementary and technically feasible alternative in middle-income contexts.

## Conclusions

Ultrasound-guided intercostal cryoanalgesia can be successfully integrated into pediatric Nuss repair in resource-limited settings. In this first Ecuadorian experience, we observed low pain scores, minimal opioid rescue, early mobilization, and short length of stay without complications. The -24-hour preoperative approach appeared to provide earlier analgesic onset in this limited experience, demonstrating procedural feasibility in a middle-income healthcare environment. Immediate preoperative (-24 hours) timing emerges as a promising option, warranting larger comparative studies.
